# Flax domestication processes as inferred from genome-wide SNP data

**DOI:** 10.1038/s41598-025-89498-9

**Published:** 2025-03-13

**Authors:** Yong-Bi Fu

**Affiliations:** Plant Gene Resources of Canada, Saskatoon Research and Development Centre, Agriculture and Agri-Food Canada, Saskatoon, SK Canada

**Keywords:** Pale flax, Cultivated flax, Indehiscent flax, Winter flax, Genotyping-by-sequencing, Plant domestication, Evolutionary genetics

## Abstract

Flax (*Linum usitatissimum* L.) is one of the founder crops domesticated for oil and fiber uses in the Near-Eastern Fertile Crescent, but its domestication history remains largely elusive. Genetic inferences so far have expanded our knowledge in several aspects of flax domestication such as the wild progenitor, the first use of domesticated flax, and domestication events. However, little is known about flax domestication processes involving multiple domestication events. This study applied genotyping-by-sequencing to infer flax domestication processes. Ninety-three *Linum* samples representing four flax domestication groups (oilseed, fiber, winter and capsular dehiscence) and its wild progenitor (or pale flax; *L. bienne* Mill.) were sequenced. SNP calling identified 16,998 SNPs that were widely distributed across 15 flax chromosomes. Diversity analysis found that pale flax had the largest nucleotide diversity, followed by indehiscent, winter, oilseed and fiber cultivated flax. Pale flax seemed to be under population contraction, while the other four domestication groups were under population expansion after bottleneck. Demographic inferences showed that five *Linum* groups carried clear genetic signals of multiple mixture events that were associated largely with oilseed flax. Phylogenetic analysis revealed that oilseed, fiber and winter flax formed two separate phylogenetic subclades. One subclade had abundant winter flax, along with some oilseed and fiber flax, mainly originating in the Near East and nearby regions. The other subclade mainly had oilseed and fiber flax originating from Europe and other parts of the world. Dating genetic divergences with an assumption of 10,000 years before present (BP) of flax domestication revealed that oilseed and fiber flax spread to Europe 5800 years BP and domestication for winter hardiness occurred in the Near East 5100 years BP. These findings provide new significant insights into flax domestication processes.

**Supplementary Information:**

The online version contains supplementary material available at 10.1038/s41598-025-89498-9.

## Introduction

Flax (*Linum usitatissimum* L.) is known to be one of the Neolithic Southwest Asian founder crops domesticated for oil and fiber uses in the Near Eastern agriculture roughly 10,000 years before present (BP)^1–4^. Genetic studies have confirmed pale flax (*L. bienne* Mill. or synonym *L. usitatissimum* L. subsp. *angustifolium* (Huds.) Thell.)^5^ as the wild progenitor of cultivated flax^6–10^. Several genetic analyses have suggested that pale flax was domesticated first for oil, rather than fiber, use^10–12^. The first archaeological finds of pale flax came from Tell Abu Hureyra in northern Syria (11200–10500 years BP)^13^ and occurred throughout the Near East by the 8th millennium BC^4^. The archaeological records from Tell Ramad in Syria (9000 years BP) revealed the first occurrence of cultivated forms of flax with an increase in seed size^2^. Twined fabrics of flax fibers found in Nahal Hemar Cave, Israel, were also dated back to 9000 years BP^14,15^. Cultivated flax then spread from the Near East to Europe and the Nile Valley^3,16^. Archaeological evidence for cultivated flax in Europe can be tracked back to 8000 years BP^17^. Archaeological finds in southwest Germany revealed larger flax seeds in the early phase of the Late Neolithic period (or 6000 years BP)^18^ and small-seeded flax 5500 years BP in the Alpine region^16^. All modern fiber varieties in use today are thought to have originated from eastern Europe^1,19,20^. The rest of the early history of flax domestication, however, remains largely elusive^4,11,21,22^.

Flax with its dual domestication purposes is unique among the founder crops domesticated in the Near East^4^ and should provide a useful model for genetic inferences of complex crop domestication processes^23–25^. Cultivated flax is known to have significant differences in many traits from its wild progenitor. Pale flax is a winter annual or perennial plant with narrow leaves and indehiscent capsules, and typically exhibit large variation in vegetative plant parts and variable growth habit^19,22,26^. In contrast, cultivated flax exhibits variable seed dormancy, large variation in generative plant parts, rapid growth, early flowering, large seeds and almost dehiscent capsules. Specifically, the major phenotypic differences exist in capsular openness, seed size, oil yield, plant stem, and winter habit of cultivated flax^26^. Interestingly, some domestication-associated traits have been used by plant researchers to group cultivated flaxes, such as indehiscent cultivated flax with a variable degree of seed capsular splitting and winter cultivated flax with a vernalization requirement^27–30^. Theoretically, these trait-specific groups of cultivated flax should carry genetic signatures of flax domestication accumulated over time. Analyzing genetic signals present in flax groups with unique domestication-associated traits^31^ should provide insights into the flax domestication processes^12,31^.

With the development of different genetic markers, genetic inferences of flax domestication history have been made over the last two decades. A marker-based analysis of different *Linum* species provided empirical evidence for the hypothesis made by Heer^6^ that pale flax is the wild progenitor of cultivated flax^9^. Several lines of genetic inference suggested that cultivated flax probably descended from a single domestication of pale flax for oil use, followed by a subsequent flax domestication process with multiple domestication events for capsular dehiscence, fiber and winter hardiness^9,12^. Also, domestication of capsular indehiscent forms occurred earlier than domestication of winter hardy forms and seemed to be simpler than domestication on winter hardiness as indehiscent cultivated flax does not have complex genetic relationships with oil and fiber flax as winter flax has^32,33^. Analyzing the genetic relationships of pale flax and cultivated flax revealed that indehiscent cultivated flax is genetically more related to pale flax^31,32^ and winter cultivated flax is closely related to oil and fiber cultivated flax^34^. Thus, these genetic inferences have advanced our knowledge in some aspects of flax domestication^33^. However, these genetic inferences generally had limited resolutions in estimations^33^ and were clouded with inadequate sampling of diverse flax^11,31^ and/or limited genomic sampling with insufficient molecular markers^12^. Important questions about flax domestication remain, such as when did cultivated flax spread to Europe? and when and where did domestication for fiber, winter hardiness, and capsular dehiscence begin? These questions can be fruitfully addressed through genetic inferences^35^, given the advances in flax genomics^36–39^.

The overall objective of this study was to infer flax domestication processes with genome-wide SNPs that were acquired through genotyping-by-sequencing. Specifically, 93 *Linum* samples representing pale flax and four domestication groups of cultivated flax (oilseed, fiber, winter and capsular indehiscence) were sequenced, genome-wide SNPs were identified by bioinformatic tools, and different genetic analyses were performed to infer flax domestication events, with the focus on when and where domestications occurred for oil, fiber, winter hardiness and/or capsular dehiscence. It was our hope that the genome-wide SNPs would carry useful genetic signals for better inferences and understanding of the various flax domestication processes.

## Materials and methods

### Plant materials and DNA extraction

The study material consisted of 93 *Linum* samples representing 91 genetically diverse *Linum* accessions originating from 32 countries, one accession duplicate and one accession biological replicate (Table 1). These accessions were selected from the flax collection maintained at Plant Gene Resources of Canada, Saskatoon, Canada, and acquired through the Standard Material Transfer Agreement (https://www.fao.org/plant-treaty/areas-of-work/the-multilateral-system/smta/en/; accessed 24 January 2025). The selected accessions represented four domestication groups of cultivated flax with major domestication-associated traits (high oil content, strong bast fiber, indehiscent capsule, and winter habit) and its wild progenitor (or pale flax). For ease of description, these four domestication groups will be named as oil, fiber, indehiscent and winter flax groups to represent oilseed, fiber flax, cultivated flax with indehiscent capsule and cultivated flax with winter habit, respectively. The selected pale flax samples represented the species distributions mainly in Turkey, Greece, Germany and France. Approximately 10 seeds were randomly chosen from each selected accession and planted in seedling trays filled with a regular soilless potting mix. Plants were grown in a greenhouse at the Saskatoon Research and Development Centre, Agriculture and Agri-Food Canada, Saskatoon, Canada, for two to three weeks for cultivated flax and up to two months for pale flax (due to vernalization requirement and slow growth). The greenhouse conditions were 22 °C during the day and 16 °C at night, with a photoperiod of 16 h between 4 am and 8 pm. Young leaf tissue from individual plants of each accession was collected, freeze-dried, and stored at − 20 °C. For this study, one individual plant was randomly selected to represent its accession, but the accession CN97473 was represented with two individual plants as two biological replicates (Table 1). Note that the accession duplicate and biological replicate were employed mainly as the control for MiSeq sequencing and for verification of intra-accession sequencing variation. DNA was extracted from 10 mg of freeze-dried flax leaf tissue using the Qiagen DNeasy Plant Mini Kit (Qiagen Inc., Toronto, ON, Canada) according to the product handbook. DNA quality was assessed using a 260/280-nm ratio from the Thermo Scientific Nanodrop 8000, and DNA was quantified by using the Invitrogen Quant-iT™ PicoGreen^®^ dsDNA Assay Kit (Life Technologies, Burlington, ON, Canada) and adjusted to 20 ng/µL with nuclease-free water.

**Table 1:** List of 93 *Linum* samples representing pale flax and four domestication groups of cultivated flax (oil, fiber, winter and indehiscent flax).

Sample			Sample sequence label	Sample label^d^	Sample			Sample sequence label	Sample label
CN^a^	Description^b^	CoO^c^			CN	Description	CoO		
107257		UNK(1)	CN107257_S1	b1	18,991	Nike	RUS	CN18991_S5	f6
19021	TMP-1191	FRA	CN19021_S2	b2	101,111	Viking	FRA	CN101111_S9	f7
107258		UNK(1)	CN107258_S15	b3	101,392	Tajga	FRA	CN101392_S10	f8
19022	TMP-1215	DEU	CN19022_S16	b4	101,397	Pskovski 2976	RUS	CN101397_S12	f9
113606	Samsun	TUR	CN113606_S15	b5	98,946	Talmune Fiber	NLD	CN98946_S15	f10
113632	Zonguldak	TUR	CN113632_S5	b6	97,325	Kotowiecki	POL	CN97325_S3	f11
107293		UNK(2)	CN107293_S6	b7	98,479	Zakar	CZE	CN98479_S16	f12
113622	Antalya	TUR	CN113622_S12	b8	98,475	Flachskopf	DEU	CN98475_S6	f13
19023	PI 254,371	UNK(3)	CN19023_S13	b9	97,871	Atlas	SWE	CN97871_S8	f14
113618	Muğla	TUR	CN113618_S2	b10	98,986	Crista	BEL	CN98986_S9	f15
113608	Samsun	TUR	CN113608_S3	b11	101,017	Baladi	CHN	CN101017_S10	f16
113640	Istanbul	TUR	CN113640_S7	b12	18,974	CDC Bethune	CAN	CN18974_S8	o1
113634	Bolu	TUR	CN113634_S8	b13	100,917	Raluga	ROM	CN100917_S9	o2
113633	Zonguldak	TUR	CN113633_S13	b14	113,643	Sari-85	TUR	CN113643_S13	o3
113623	Antalya	TUR	CN113623_S14	b15	52,732	Norlin	CAN	CN52732_S14	o4
113610	Denizli	TUR	CN113610_S2	b16	33,399	Bison	USA	CN33399_S13	o5
113638	Çanakkale	TUR	CN113638_S4	b17	19,003	AC McDuff	CAN	CN19003_S16	o6
113297	Island of Koss	GRC	T19717_S5	b18	18,989	Atalante	FRA	CN18989_S1	o7
113637	Bursa	TUR	CN113637_S7	b19	101,265	Amason	GBR	CN101265_S7	o8
113635	Bolu	TUR	CN113635_S8	b20	101,174	Rastatter	DEU	CN101174_S9	o9
113641	Çanakkale	TUR	CN113641_S9	b21	101,292	Zarjanka	RUS	CN101292_S10	o10
113636	Bilecik	TUR	CN113636_S10	b22	101,171	Hermes	FRA	CN101171_S1	o11
113603	Samsun	TUR	CN113603_S11	b23	100,832	Barbarigo	CZE	CN100832_S6	o12
113630	Kastamonu	TUR	CN113630_S12	b24	101,268	Raisa	NLD	CN101268_S1	o13
113627	Sinop	TUR	CN113627_S13	b25	97,888	Tomagoan	IRN	CN97888_S6	o14
113626	Samsun	TUR	CN113626_S1	b26	101,614	Signal	BLR	CN101614_S14	o15
113617	İzmir	TUR	CN113617_S2	b27	101,233	Rolin	ROM	CN101233_S15	o16
113628	Karabük	TUR	CN113628_S3	b28	98,256	Arreveti	IND	CN98256_S4	o17
113620	Muğla	TUR	CN113620_S12	b29	97,436	Giza	EGY	CN97436_S7	o18
113298	Island of Evia	GRC	T19718_S13	b30	101,245	Bryta	POL	CN101245_S11	o19
113629	Kastamonu	TUR	CN113629_S16	b31	101,237	Artemida	LTU	CN101237_S14	o20
97606	PI 522,771	ESP	CN97606_S3	d1	98,178	1285-S	AFG	CN98178_S10	w1
100852*	Grandal	PRT	CN100852_S4	d2	97,004	PI 196,003	JPN	CN97004_S11	w2
98833	PI 524,102	BDI	CN98833_S5	d3	96,960	PI 181,774	SYR	CN96960_S12	w3
97605	PI 522,770	BDI	CN97605_S6	d4	97,205	Redwing 92	GRC	CN97205_S1	w4
100837	LIN-1193	TUR	CN100837_S7	d5	97,756	Italia Roma	ARG	CN97756_S2	w5
100910*	Grandal	PRT	CN100910_S11	d6	96,846	Bujumbura	BDI	CN96846_S3	w6
97769	Abertico	PRT	CN97769_S14	d7	98,283	La Previzion	HUN	CN98283_S4	w7
97473**	PI 522,638	BDI	CN97473_S8	d8	98,509	PI 523,675	ISR	CN98509_S5	w8
97768	Mourisco, E730	PRT	CN97768_S16	d9	97,009	Beladi Y 6903	EGY	CN97009_S6	w9
101424	Torzhokshij 4	RUS	CN101424_S5	d10	97,102	PI 250,561	PAK	CN97102_S7	w10
97473**	PI 522,638	BDI	CN97473_S15	d11	96,915	Uruguay 36/49	AUS	CN96915_S3	w11
101086	Ariadna	HUN	CN101086_S12	f1	100,829	LIN-255	TUR	CN100829_S4	w12
98935	Motley Fiber	BLR	CN98935_S2	f2	100,828	LIN-1260	TUR	CN100828_S4	w13
101405	Mures	ROM	CN101405_S11	f3	96,848	PI 165,006	TUR	CN96848_S11	w14
101160	Wiko	AZE	CN101160_S14	f4	96,902	PI 175,767	TUR	CN96902_S16	w15
101388	Saskai	CZE	CN101388_S15	f5					

^a^CN = Canadian National accession number at Plant Gene Resources of Canada, Saskatoon, Canada (https://pgrc-rpc.agr.gc.ca/gringlobal/search; accessed 24 January 2025). * and ** show accession duplicates and biological replicates, respectively.

^b^Description of an accession includes the record for varietal or local name, location, and feature.

^c^CoO = Country of origin, following ISO 3166-1 alpha-3 country code. UNK = unknown origin, but the seed source is shown with a number in parentheses: 1, Jardin Botanique de la Ville et de l’Universite de Caen, France; 2, All-Russian Flax Research Institute, VNIIL, Torzhok, Russia; 3, Indian Agricultural Research Institute, Dehli, India.

^d^Sample follows its *Linum* group label (five letters (b, d, f, o, w) for pale flax, indehiscent, fiber, oil, winter cultivated flax, respectively) plus the numbering of the sequenced samples (S) in a sequencing run.

### Genotype-by-sequencing

Six sequencing libraries of up to16 samples each were prepared following the genetic diversity-focused GBS (gd-GBS) protocol described by Peterson et al.^40^. Briefly, each library preparation started with the digestion of 200 ng of purified genomic DNA with the restriction enzyme combination: *Pst*I and *Msp*I (New England Biolabs, Whitby, ON, Canada). Custom adapters were ligated onto the 5′ and 3′ ends of the restriction fragments, which were then purified using 1 × Agencourt AMPure XP Beads (Beckman Coulter, Mississauga, ON, Canada) and amplified with PCR to add multiplexing indexes and Illumina (San Diego, CA, USA) specific binding sites. The amplified products were quantified, concentrated, and pooled into groups of four samples prior to size selection for fragments with insert sequences of 250–450 bp using the Pippin Prep instrument (Sage Science, Beverly, MA, USA). Each group of four samples was quantified using an Invitrogen Quant-iT™ PicoGreen^®^ dsDNA Assay Kit (Life Technologies, Burlington, ON, Canada) and combined in equimolar amounts, resulting in six multiplexed DNA libraries of up to 16 samples each. Sequencing was performed over six runs at the Saskatoon Research and Development Centre using an Illumina MiSeq instrument with a MiSeq Reagent Kit v3 (600 cycles) and paired-ends of 250 bp in length. The raw sequences were acquired in April and May 2014 and deposited into NCBI’s SRA database under BioProject ID of PRJNA1106517 in April 2024.

### Sequence alignment and SNP calling

A pair of demultiplexed FASTQ files were generated for each sample: one in the forward direction and another in the reverse direction. FastQC (Babraham Bioinformatics; https://www.bioinformatics.babraham.ac.uk/projects/fastqc/; accessed 19 November 2024) was applied to assess the overall sequencing quality of each sample. FASTQ files were trimmed with Trimmomatic v0.32^41^ to remove any adapter sequences, trim low quality sequence (below a Phred score of 24), and remove any sequences shorter than 80 bases. The following trim settings were applied: ILLUMINACLIP: TruSeq3-PE-2.fa; SLIDINGWINDOW:10:24; and MINLEN:80. FastQC was run again to verify the Illumina adapter sequences were removed. A reference genome sequence was obtained from the genome assembly of the fiber flax cultivar YY5 (lus.final.fasta.gz)^39^, as this assembly was slightly better than the original assembly of oilseed flax CDC Bethune in length and quality^37,42^. The samples were aligned against the reference genome sequence using the Burrows-Wheeler Aligner v0.7.17^43^ with BWA-MEM algorithm. The resulting BAM files were filtered to remove PCR duplicates using the MarkDuplicates tool from the Genome Analysis Toolkit v4.2.6.1 (GATK)^44^. Samtools v1.6^45^ sort option was applied to produce sorted BAM files. Table S1 summarizes the number of mapped reads in the sorted BAM file for each sample. Single nucleotide polymorphisms (SNPs) and genotypes were generated using ANGSD v 0.921^46^ based on the sorted BAM files and the reference genome of YY5.

### Genetic diversity analysis

To estimate nucleotide diversity in the *Linum* samples, ThetaD statistics and Tajima’s *D* tests for neutrality were generated using ANGSD with an empirical Bayes approach^46^ for each chromosome using 50 kb non-overlapping sliding windows with steps of 10 kb. This was done for all 93 samples and for the five *Linum* groups. To assess the genetic associations of the assayed samples, a principal component analysis was conducted using the SNPRelate Bioconductor R package^47^ based on the SNP VCF file. Extra effort was made to perform an analysis of molecular variance (AMOVA) on the acquired SNP genotypes using Arlequin v 3.1^48^. The AMOVA analysis generated the Phi statistics as measures of inter-group genetic distances among the five *Linum* groups, based on which a neighbor-joining tree of the five *Linum* groups was generated using NTSYS-pc 2.1^49^.

### Phylogenetic analysis

Phylogenetic analysis of the 93 *Linum* samples was performed using BEAST v2.7.6 software^50^ and RAxML^51^ to infer the phylogenetic tree, and SplitsTree 4.0 software^52^ to infer the phylogenetic network. For inference of the phylogenetic tree, we first manually converted haplotype SNP FASTA data generated by ANGSD to NEXUS format, followed by testing and selecting tree construction options for the use of BEAST software. These training tests suggested the use of the following settings: Gamma site model with *HKY* substitution model; clock model with *optimized relaxed clock*; and tree prior with *Coalescent Constant Population.* The rest of the options were kept with default values. The output tree files were checked for convergence with Tracer v1.7.2 and loaded into TreeAnnotator v2.7.6 in the BEAST package with default options to combine and construct a maximum clade credibility (MCC) tree. The MCC tree was visualized using Figtree_v1.4.5 software (http://tree.bio.ed.ac.uk/software/figtree/; accessed 19 November 2024) to display different versions of the MCC tree with posterior probability, node age, and height_95%_HPD. To date the nodes, the root node was assumed to the age of 10,000 years and the standard deviation of a node age is based on height_95%_HPD and scaled to the root age. Note that the assumed root age of 10,000 years for oil flax was within the 10,000 to 12,000 years of the other Neolithic Southwest Asian founder crops like wheat and barley in the Near East^4^. Comparisons were also made among MCC trees, RAxML-based maximum likelihood phylogenetic trees, and phylogenetic networks generated by SplitsTree to evaluate the consistency of the revealed phylogenetic signals.

### Demographic inference

Inferences of population mixtures among the five *Linum* groups were made using Admixtools2^53^, TreeMix^54^ and OptM^55^. Admixtools2 is an R package with new, fast implementations of the core Admixtools programs^53^. Thus, a custom R script was written to input genotype data in PLINK format that was converted by VCFtools^56^ from the SNP VCF file and to implement various Admixtools functions to find and plot the mixture graph. For the application of function find_graphs, pale flax was used as outpop and admixture events ranged from 2 to 10. TreeMix is a method for inferring the patterns of population splits and mixtures in the history of a set of populations from genome-wide allele frequency data. For this study, a shell script was written following TreeMix’s instructions to run migration edges from 1 to 10 with pale flax as root. Its input allele frequency data was generated from the genotype data in Microsoft Excel following TreeMix’s instructions on input data format. OptM is a method of estimating an optimal value of migrations for TreeMix based on the second-order rate of change in likelihood across incremental values of migrations. It is implemented with two R functions: optM.R and plot_optM.R. For this study, the original shell script for TreeMix was modified to allow for multiple iterations following OptM’s instructions and optM.R was modified for proper data inputs. Both Evanno and linear optimization options were applied.

Inferences of splitting time between two *Linum* groups were also made using SMCPP software^57^ with the assumption that no gene flow occurred after two groups split. Four pairs of *Linum* groups (pale vs. oil flax, oil vs. indehiscent flax, oil vs. fiber flax, and oil vs. winter flax) were analyzed for each chromosome. Specifically, a total of 60 SMCPP runs (4 group pairs × 15 chromosomes) were made and each run was executed using a custom shell script involved with four SMC++ commands (vcf2smc, estimate, split and plot) with data inputs of the SNP VCF file, group sample labels, chromosome length, mutation rate of 4.86e-8 and timepoints 1 to 10,000 (generations). The mutation rate was acquired from an unpublished study of deleterious base-substitution mutations in 70 flax samples and it is close to those mutation rates estimated in four selfing crops^58^.

## Results

### SNP identification

Six MiSeq sequencing runs generated an average of 1.4 million FASTQ paired raw sequence reads per sample and a range of 0.16 to 4.00 million mapped reads in the BAM file per sample with an average of 2.1 million mapped reads (Table S1). There were only 9 (out of 93) samples with mapped sequence reads of one million or fewer. The SNP calling with ANGSD from mapped sequence reads of the assayed 93 samples generated 28,331 SNPs without missing values across the 15 chromosomes (28,242 SNPs) and two scaffolds (89 SNPs). Removing SNPs on scaffolds and SNPs with derived alleles of frequency 0.0499 or smaller (or with derived alleles present in five or fewer samples) generated 16,998 SNPs on the 15 chromosomes for further analysis. Comparing SNP calls between two accession duplicates and between two biological replicates revealed 1168 and 418 (or 6.9% and 2.5% out of 16,998) SNPs with mismatch, respectively. These differences could reflect intra-accession variations and technical errors from sequencing and bioinformatic analyses. However, such extents of SNP mismatch could be expected for an intra-accession SNP variation alone, as up to 6% outcrossing rate was reported for fiber flax^59^. Thus, the duplicate and replicate samples were not excluded and treated as separate samples for further analyses below. Further assessments of 16,998 SNPs revealed their wide distributions across the 15 chromosomes for all 93 samples and for each group of pale flax and cultivated flax, as illustrated in Table S2. The allelic frequency distributions for the 16,998 SNPs in the 93 samples and five *Linum* groups are displayed in Figure S1. A majority of alleles had frequencies of 0.8 or larger, except in the 11 samples of indehiscent flax.

### Nucleotide diversity

The nucleotide diversity was estimated across 15 chromosomes for the five *Linum* groups (Table 2). It is clear that the nucleotide diversity in terms of ThetaD per site varied among the chromosomes for any *Linum* group. For pale flax, the diversity estimates ranged from 0.00183 (Chromosome 1) to 0.00254 (Chromosome 8) and averaged 0.00218. Similarly, oil flax had nucleotide diversity ranging from 0.00062 (Chromosomes 10 and 14) to 0.00114 (Chromosome 11) and averaging 0.00091. Ranking the five *Linum* groups based mean nucleotide diversity estimates revealed that pale flax had the largest mean nucleotide diversity (0.00218), followed by indehiscent flax (0.00164), winter flax (0.00110), oil flax (0.00091) and fiber flax (0.00074).

**Table 2:** Nucleotide diversity and Tajima’s *D* test for neutrality across 15 flax chromosomes (chr) for five *Linum* groups (pale flax, indehiscent flax, oil flax, fiber flax and winter flax).

Chr	Nucleotide diversity (π per site)					Tajima’s D test				
	Pale	Indehiscent	Oil	Fiber	Winter	Pale	Indehiscent	Oil	Fiber	Winter
1	0.00183	0.00168	0.00089	0.00069	0.00110	− 0.351	0.112	− 0.391	− 0.886	− 0.381
2	0.00230	0.00180	0.00073	0.00063	0.00091	0.244	0.004	− 0.615	− 0.853	− 0.640
3	0.00199	0.00157	0.00084	0.00063	0.00110	− 0.220	− 0.377	− 0.422	− 1.030	− 0.380
4	0.00240	0.00147	0.00101	0.00074	0.00128	0.123	− 0.041	− 0.243	− 0.821	− 0.245
5	0.00240	0.00161	0.00108	0.00087	0.00129	0.243	0.073	− 0.157	− 0.567	− 0.255
6	0.00215	0.00150	0.00087	0.00068	0.00102	0.034	− 0.189	− 0.362	− 0.878	− 0.556
7	0.00214	0.00172	0.00103	0.00078	0.00128	− 0.045	− 0.006	− 0.270	− 0.743	− 0.212
8	0.00254	0.00181	0.00099	0.00079	0.00123	0.315	− 0.023	− 0.281	− 0.654	− 0.356
9	0.00219	0.00155	0.00081	0.00070	0.00091	0.073	− 0.005	− 0.503	− 0.638	− 0.722
10	0.00188	0.00142	0.00062	0.00056	0.00078	− 0.347	− 0.305	− 0.849	− 0.983	− 0.897
11	0.00217	0.00183	0.00114	0.00090	0.00133	− 0.080	0.125	− 0.085	− 0.487	− 0.295
12	0.00192	0.00156	0.00082	0.00069	0.00100	− 0.246	− 0.205	− 0.389	− 0.825	− 0.539
13	0.00221	0.00172	0.00104	0.00094	0.00127	− 0.087	− 0.038	− 0.102	− 0.378	− 0.186
14	0.00225	0.00167	0.00062	0.00053	0.00068	0.231	− 0.042	− 0.774	− 1.017	− 1.108
15	0.00234	0.00173	0.00113	0.00091	0.00131	0.235	− 0.024	− 0.029	− 0.317	− 0.183
Mean	0.00218	0.00164	0.00091	0.00074	0.00110	0.008	− 0.063	− 0.365	− 0.738	− 0.464
SD	0.00021	0.00013	0.00017	0.00013	0.00021	0.226	0.145	0.242	0.225	0.278

SD is standard deviation.

Evaluating Tajima’s *D* tests for neutrality revealed that pale flax and indehiscent flax had positive *D* values for eight and four chromosomes, respectively, while the other three *Linum* groups had negative *D* values for all 15 chromosomes (Table 2). By averaging across the chromosomes, pale flax had an overall positive *D* value of 0.008, while the other four groups had negative *D* values ranging from − 0.738 (fiber flax) to − 0.063 (indehiscent flax). These *D* values (Table 2), however, were smaller than an absolute value of 2 and thus were not statistically significant from zero at *P* < 0.05. In spite of the non-significant tests, the results suggested that pale flax was under population contraction, while the other four domestication groups were under population expansion after bottleneck.

### Genetic structure and relationship

**Fig. 1:**
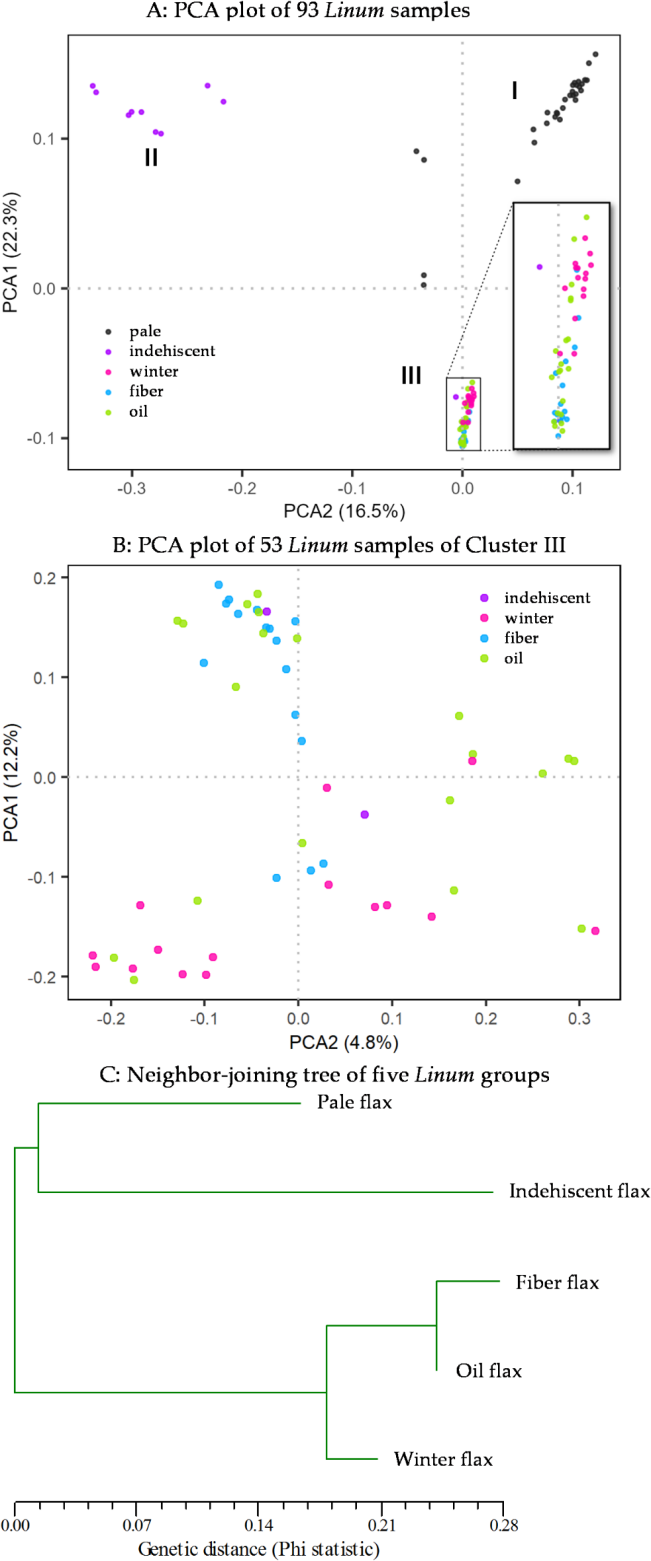
Genetic associations of 93 *Linum* samples as revealed by the plots from the principal component analysis (PCA) (**A** and** B**) and the neighbor-joining tree (**C**) of pale flax and four domestication groups of cultivated flax. Panel **A** shows three major clusters of 93 samples as I, II and III. Panel **B** displays the PCA plot of 53 *Linum* samples of Cluster III in Panel **A** from a separate analysis of their SNP data alone. Panel **C** is based on Phi statistics acquired from the analysis of molecular variance on five *Linum* groups.

The principal component analysis revealed that there were three major genetic clusters present in the 93 samples (Fig. 1A). Pale flax and indehiscent flax had their own clusters (Cluster I and Cluster II, respectively), while the third cluster (or Cluster III) largely consisted of oil, fiber and winter flax and had two samples of indehiscent flax. Note that the first two principal components explained 22.3% and 16.5% variances. A separate PCA of the 53 samples of Cluster III revealed more detailed, but complex, associations of these genetically closed samples (Fig. 1B). There were no clear separations among oil, fiber and winter flax. Fiber flax and winter flax were genetically close to oil flax, but relatively not so close to each other (Fig. 1B). These genetic associations were consistent with the neighbor-joining tree of the five *Linum* groups, which was obtained based on the Phi statistics obtained from the AMOVA analysis (Fig. 1C). Oil flax and fiber flax were genetically more related and they formed a group with winter flax, as illustrated in Cluster III of Fig. 1A. In contrast, pale flax and indehiscent flax were genetically closer to each other than to oil flax.

### Phylogenetic inference

**Fig. 2:**
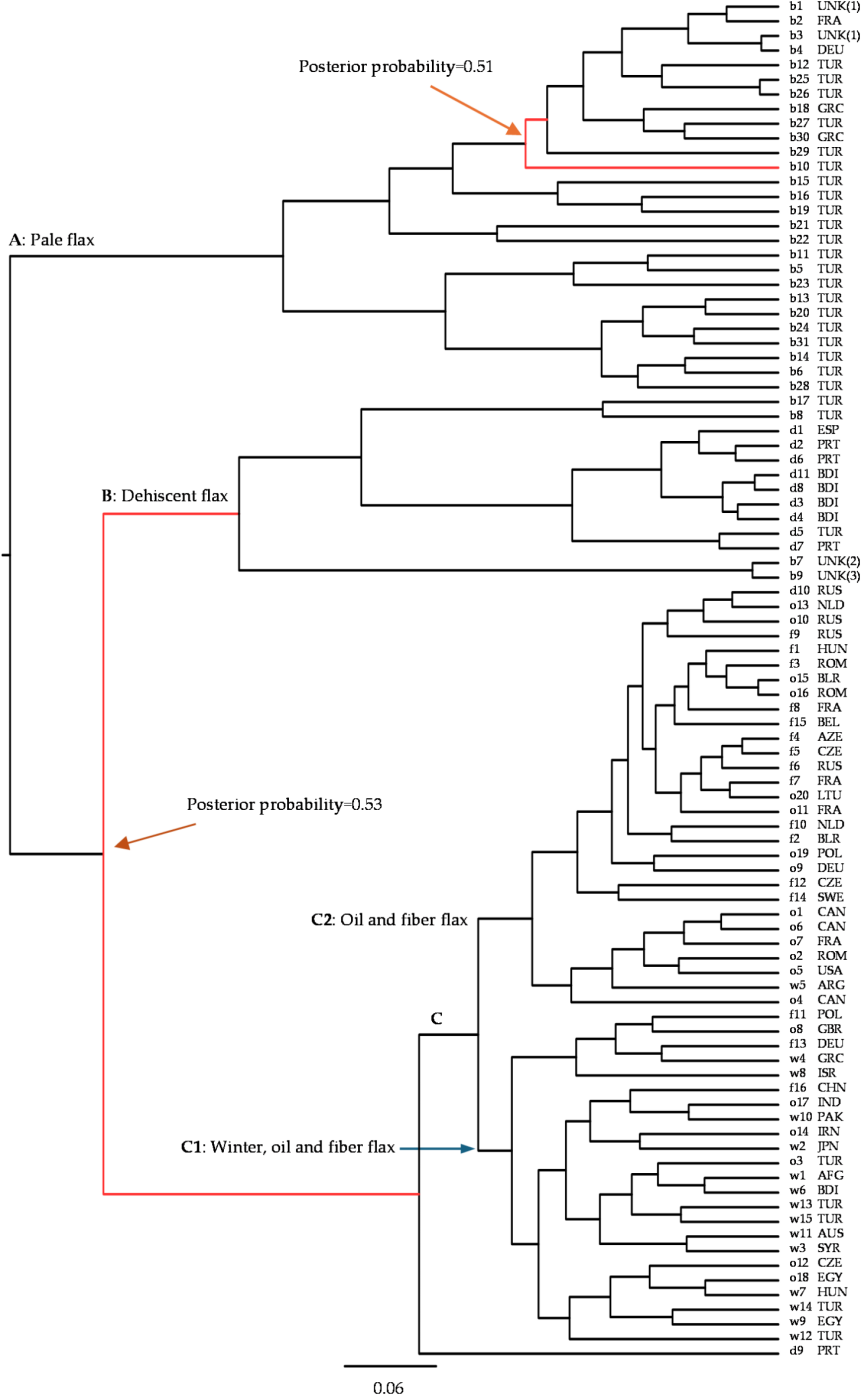
The maximum clade credibility tree of 93 *Linum* samples representing five *Linum* groups (b = pale flax, o = oil flax, f = fiber flax, w = winter flax, and d = indehiscent flax) obtained by the BEAST program. Three major clades (**A**–**C**) are labeled and clade **C** is further divided into two (**C**1 and **C**2). Two nodes with the posterior probability of 0.90 or lower are highlighted in red. Sample label and its country origin (see Table 1) are shown.

The BEAST-based phylogenetic analysis revealed a Bayesian MCC tree of the 93 *Linum* samples (Fig. 2), which has three major clades with branch supports. Clade A had only pale flax with 27 samples. Clade B had nine indehiscent flax samples and four pale flax samples, representing mainly indehiscent flax. Clade C had all 20 oil, 16 fiber and 15 winter flax samples, along with two indehiscent flax samples, and it can be further divided into two subclades. Clade C1 had 14 winter, 6 oil, and 3 fiber flax samples. There were two major features in clade C1. First, seven (out of 14) winter flax samples were closely related to three (out of six) oil flax samples in the Near East. Second, two fiber flax samples from Germany (f13) and Polland (f11) were genetically close to two winter flax samples from Greece (w4) and Israel (w8). Clade C2 had 14 oil, 13 fiber, 1 winter and 1 indehiscent flax samples. Note that the only winter flax sample in clade C2, w5, was collected from Argentina but descended from the cultivated flax named “Italia Roma” which was presumably developed and grown in Italy (see Table 1). The same patterns of *Linum* divergence were found from the phylogenetic tree generated by the RAxML program (Figure S2) and the NeighborNets obtained by the SplitsTree4 program (Figure S3). It is more obvious that the same patterns of divergence were revealed for oil, fiber and winter flax samples. However, the RAxML-based tree had more information on the divergence of pale flax and the NeighborNets displayed more information on the networks of pale flax and dehiscent flax.

**Fig. 3:**
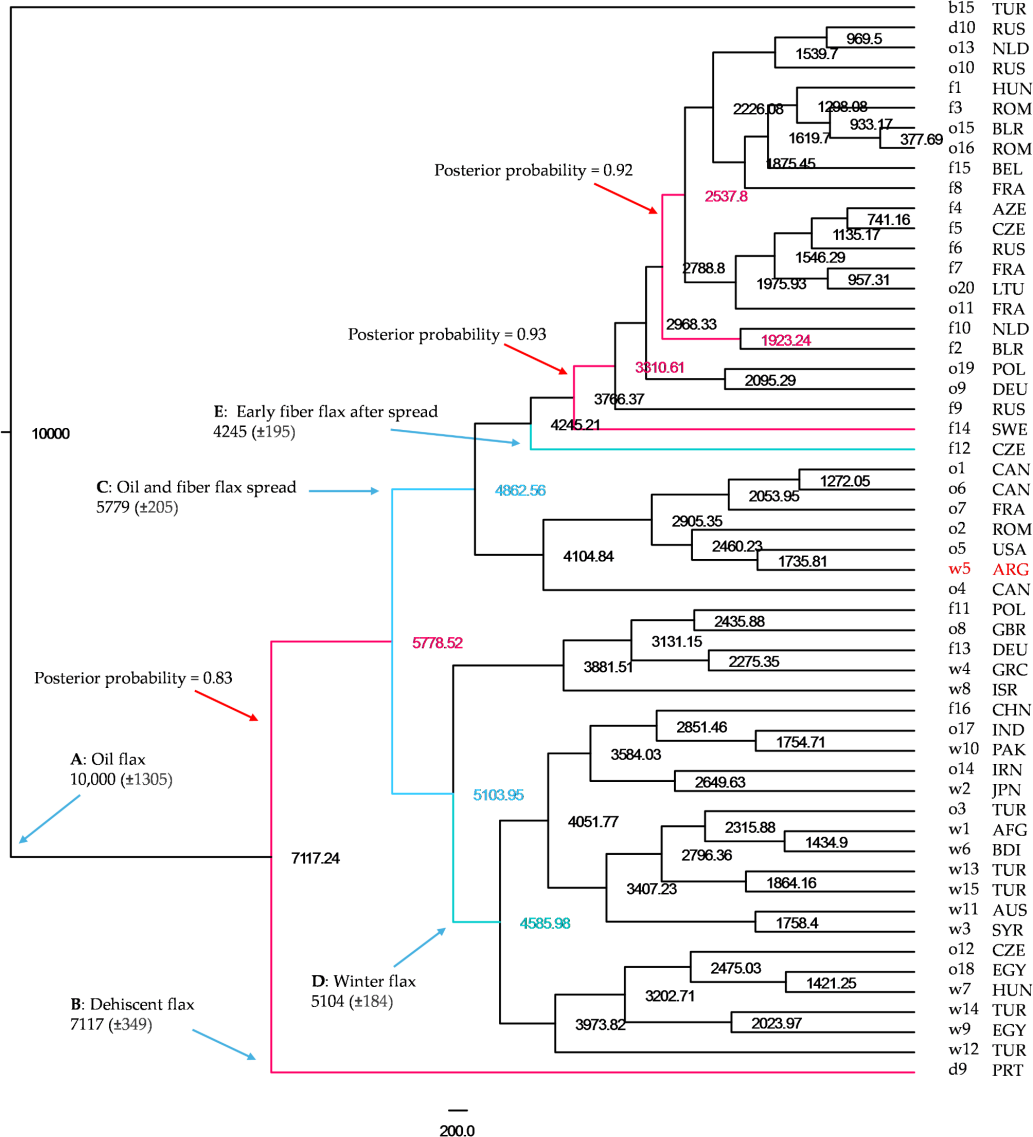
The maximum clade credibility trees of 54 selected *Linum* samples representing pale flax and four domestication groups of cultivated flax obtained by the BEAST program. Node ages relative to a root age of 10,000 years assumed for flax domestication start from pale flax are shown, along with the scale bar in years. The five major divergences representing flax domestication (from** A** to** E**) are indicated in blue narrow, along with the node ages (and their standard deviations). Three nodes with the posterior probabilities of 0.83 to 0.93 are highlighted in red. Sample label and its country of origin (see Table 1) are shown. The sample w5 collected in 1939 from Argentina had the pedigree of Italia Roma/CI 1005-2 and thus was a descendent of a flax cultivar grown in Italy.

Extra effort was also made to date nodes of the MCC tree of 54 *Linum* samples generated with the same BEAST model and priors with the pale flax sample b15 as an outgroup (Fig. 3). The new MCC tree had nearly all of the nodes with posterior probabilities of 1 except three nodes with the posterior probabilities of 0.83 to 0.93. Rooted to the pale flax sample b15 with 1 × 10,000 years for flax domestication for oil (node A), indehiscent flax (node B) was estimated to occur 7117 years ago (with a standard deviation of 349 years). Oil flax and fiber flax (node C) spread to Europe and other regions 5779 years BP (with a standard deviation of 205 years). Note that, when the five non-European samples (o1, o4, o5, o6 and w5) were excluded from node C in a separate BEAST-based dating analysis, node C was dated 5939 years BP (with a standard deviation of 305 years). The node largely representing winter flax (node D) was dated 5104 years BP (with a standard deviation of 184 years). The earliest fiber flax that can be directly dated (node E) was sample f12 from Czech with 4245 years BP, along with a standard deviation of 195 years. To understand the dating uncertainties, Figure S4 was generated to illustrate the MCC tree with its node height and node height_95%_HPD estimates.

### Demographic inference

**Fig. 4:**
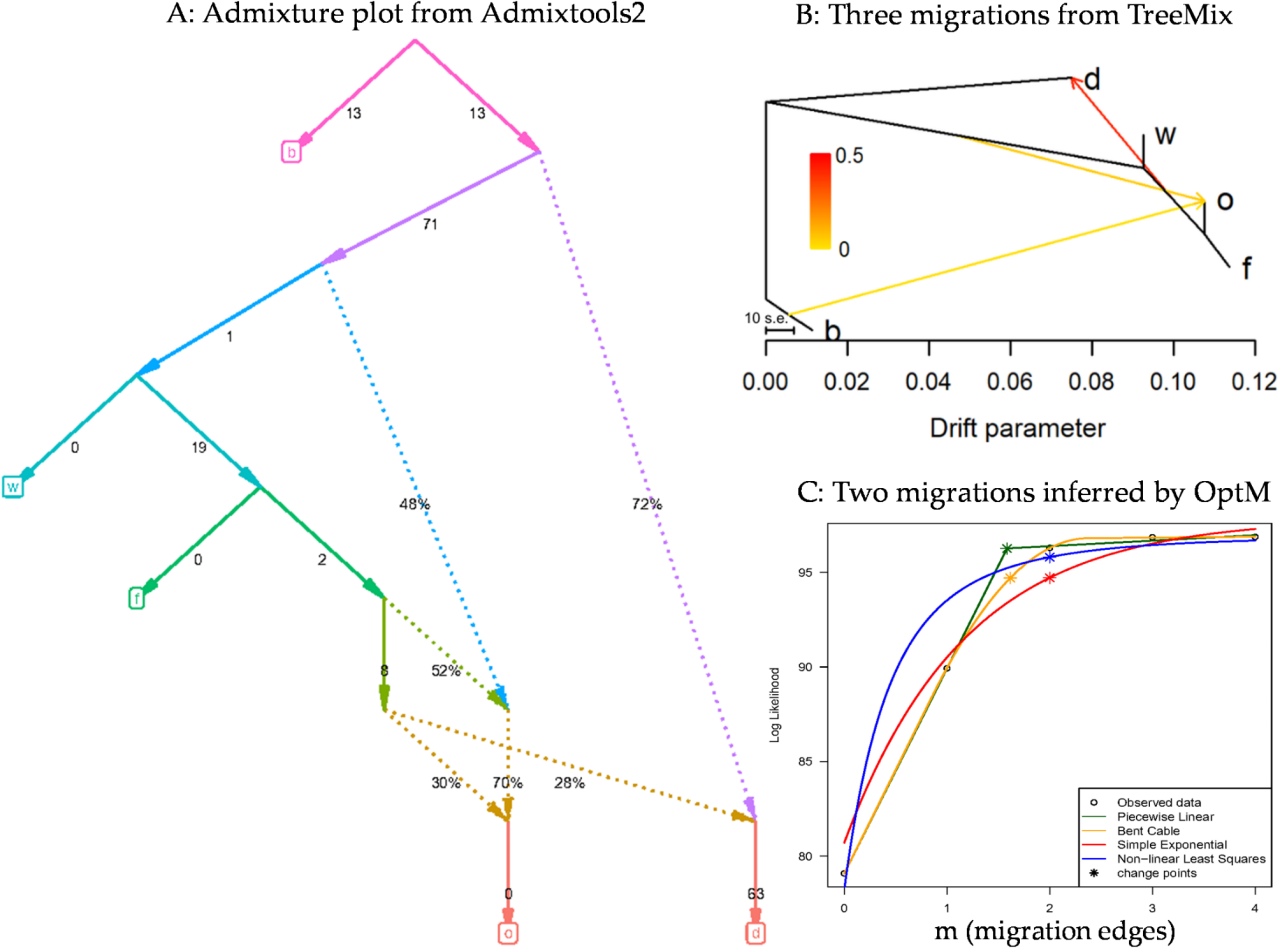
Demographic inferences of five *Linum* groups (b = pale flax, o = oil flax, f = fiber flax, w = winter flax, and d = indehiscent flax) with multiple mixture events, inferred by Admixtools2 (**A**), TreeMix (**B**), and by OptM (**C**) with the linear fitting of the optimal mixture events.

The demographic inferences of pale flax and four domestication groups of cultivated flax by three different tools revealed multiple mixture events, as illustrated in Fig. 4. Specifically, Admixtools2 revealed multiple mixture events, particularly toward oil and indehiscent flax (Fig. 4A). The TreeMix-based analysis identified three migrations, two of which were toward the oil flax (Fig. 4B). More information on the inferences of these migrations can be extracted from the detailed results of the TreeMix-based analysis with migration edges from 1 to 10, as illustrated in Figure S5. However, the OptM-based analysis with the linear optimization option confirmed only two migrations in the assayed *Linum* samples (Fig. 4C). These results together indicated that multiple migrations occurred among the five *Linum* groups.

**Fig. 5:**
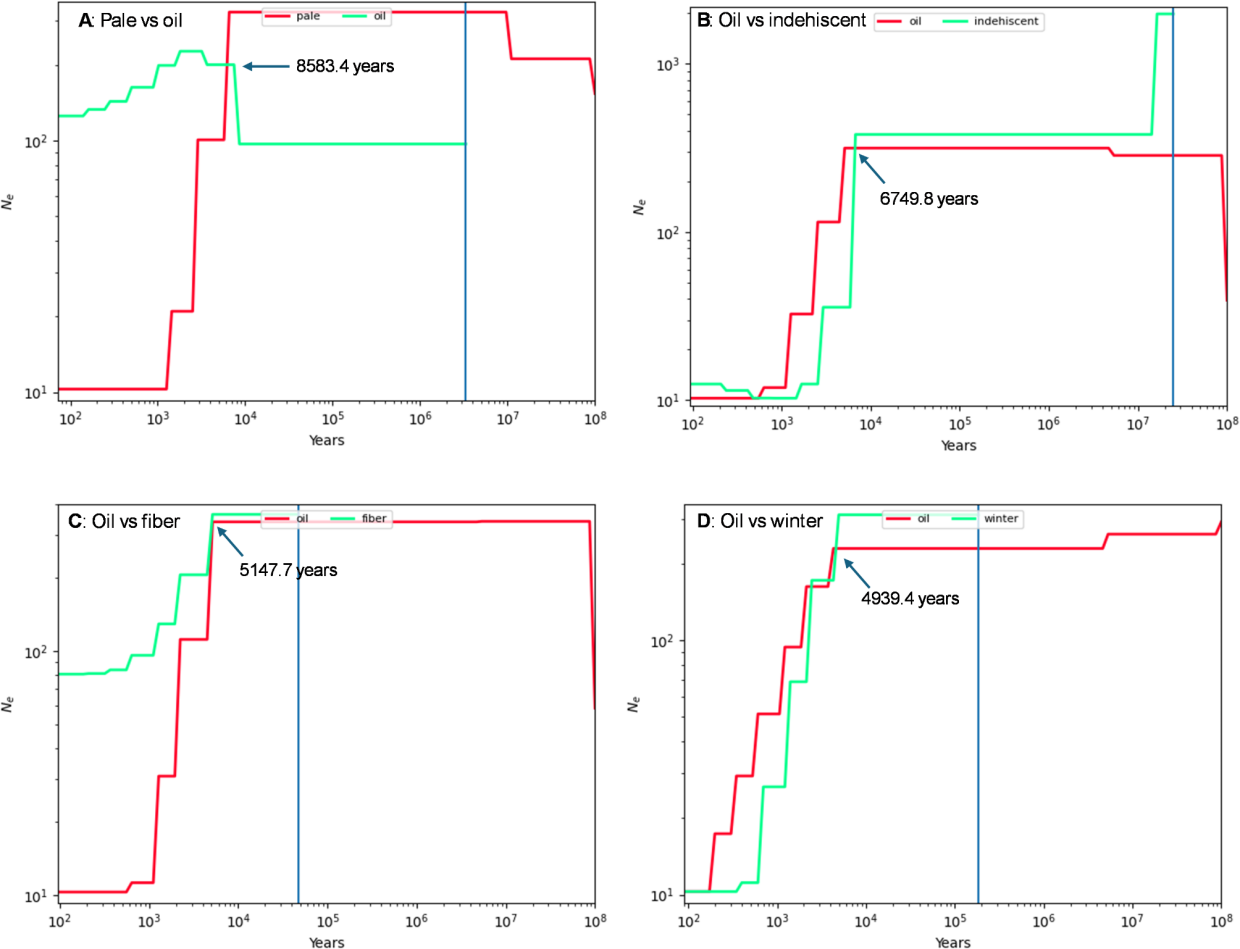
Illustrations with selective results of the demographic inferences of the domestication split times (in years) for four group pairs of pale flax and cultivated flax using SMCPP software, assuming the start at 10,000 years BP. Two groups were split when their effective population sizes (*N*_e_) were crossed over time. Panel (**A**) shows oil flax was split from pale flax 8583.4 years BP based on chromosome 14. Panel (**B**) displays the splitting of indehiscent flax from oil flax 6749.8. years BP based on chromosome 15. Panel (**C**) demonstrates fiber flax was split from oil flax 5147.7 years BP based on chromosome 15. Panel (**D**) reveals the splitting time between oil flax and winter flax was 4939.4 years BP based on chromosome 3. Note that the complete set of results on split time estimates is shown in Table 3.

The demographic inferences of splitting time between any two *Linum* groups through the SMCPP analysis produced the estimates of splitting time (in years) of four group pairs of pale flax and cultivated flax: pale vs. oil, oil vs. indehiscent, oil vs. fiber, and oil vs. winter (Table 3). The estimates varied greatly across 15 chromosomes for each pair and some estimates were not obtainable for some chromosomes due to non-convergency of estimation. Based on the estimates averaged across the chromosomes, oil flax split from pale flax 7461 years BP (with a range of 5027 to 12,092 years). Indehiscent flax departed from oil flax 7093 years BP (with a range of 4500 to 9483 years). Fiber flax diverged from oil flax 5407 years BP (with a range of 2327 to 8607 years). Winter flax separated from oil flax 4905 years BP (with a range of 3676 to 7410 years). To aid with the understanding of these estimates of splitting time, Fig. 5 illustrates the demographic inferences of splitting time for each pair from one chromosome. For example, Fig. 5A displays the divergence of oil flax from pale flax 8583 years BP, based on the genetic signals at chromosome 14.

**Table 3:** Results of demographic inferences of splitting time (in years) across 15 flax chromosomes for four group pairs of pale flax and cultivated flax using SMCPP software, assuming the domestication start at 10,000 years BP.

Chromosome	Pale-Oil	Oil-Indehiscent	Oil-Fiber	Oil-Winter
1	5,074.4	8,707.5	5,035.3	5,110.4
2	10,010.5	7,436.3	8,606.7	3,712.0
3	5,029.4	4,886.4	4,935.9	4,939.4
4	NC	8,699.8	4,197.7	5,700.5
5	NC	6,111.4	5,257.3	5,270.0
6	5,026.6	7,371.4	4,931.3	5,076.3
7	5,581.8	4,857.6	NC	7,410.0
8	NC	9,483.1	NC	NC
9	NC	9,449.4	2,326.8	4,005.6
10	10,064.8	7,590.9	NC	NC
11	12,092.3	4,500.2	NC	3,806.0
12	5,274.2	5,218.7	NC	NC
13	5,149.3	6,777.0	5,087.9	5,043.7
14	8,583.4	8,559.1	8,539.3	3,676.1
15	10,180.6	6,749.8	5,147.7	5,105.5
Mean	7,460.7	7,093.2	5,406.6	4,904.6
Standard deviation	2,730.9	1,697.3	1,879.0	1,049.8
Minimum	5,026.6	4,500.2	2,326.8	3,676.1
Maximum	12,092.3	9,483.1	8,606.7	7,410.0

NC = not convergent.

## Discussion

Our genetic inferences of pale flax and four domestication groups of cultivated flax based on genome-wide SNP data revealed a novel set of interesting findings on flax domestication. First, pale flax had the largest nucleotide diversity, followed by indehiscent, winter, oil and fiber cultivated flax. Pale flax seemed to be under population contraction, while the other four domestication groups were under population expansion after bottleneck. Second, these five *Linum* groups had clear genetic signals of multiple mixture events that were more associated with oil flax. Third, oil, fiber and winter flax formed two separate phylogenetic clades. One clade consisted of all three domestication groups, but predominantly winter flax, and these samples mainly originated in the Near East and nearby regions. The other clade mainly had oil and fiber flax originating from Europe and other parts of the world. Fourth, oil and fiber flax were dated to spread to Europe 5800 years BP and domestication for winter hardiness occurred in the Near East 5100 years BP. These findings provide new significant insights into flax domestication processes.

The results of Tajima’s *D* test with implication of population contraction and/or expansion among the *Linum* groups are novel, but largely expected only for cultivated flax groups, as cultivated flax was under artificial selection by humans for traits adaptable to new environments for over 10,000 years. It is possible that the poor geographical coverage of the assayed pale flax samples, as discussed below, had contributed to the finding of population contraction in pale flax. Similarly, the revealed patterns of nucleotide diversity among the five *Linum* groups (Table 2), in which cultivated flax had lower nucleotide diversity than pale flax, and the genetic divergences among the five *Linum* groups (Fig. 1) were compatible with those from previous genetic diversity studies based on other types of genetic markers^11,31,34^. Also, the finding of multiple mixture events among the *Linum* groups (Fig. 4) was consistent with the report of flax latitudinal adaptation through post-domestication gene flow from wild to domestic species^20^. Together, these findings provide the genetic evidence that flax domestication had different genetic impacts on the assayed *Linum* groups.

The phylogenetic analysis revealed an interesting finding of two subclades for cultivated flax with two unique features (Fig. 3). First, one subclade had cultivated flax samples (mainly of oil and fiber flax) originating from Europe and other parts of the world. This subclade was unique in sample origin and had no samples originating from the Near East. This feature provided genetic evidence that oil and fiber flax spread north to Europe and then to other parts of the world. Second, the other subclade had 23 samples mixed with oil, fiber and winter flax. Its unique feature was the dominance of winter flax with 14 samples, of which seven samples originated from the Near East, along with three (out of six) oil flax samples also originating from the Near East. This feature suggested that the initial domestication for winter hardiness occurred in the Near East, likely for better growth of oil flax.

There was another interesting finding from the phylogenetic inferences (Fig. 2, S2 and S3): there is no specific clade or branch predominantly composed of fiber flax. There were 13 fiber flax samples intermingled with 14 oil flax samples in subclade C2 (Fig. 2) and there were two fiber flax samples from Germany and Poland that had close genetic relations with two winter flax samples from Greece and Israel in subclade C1. These phylogenetic relations of fiber flax intermingled with oil and winter flax did not provide convincing support for the previous notion that all modern fiber varieties in use today have originated from eastern Europe^1,19,20^. This finding also revealed difficulties in the genetic inferences of domestication for fiber. There were no clear genetic signals as to when and where domestication for fiber started and when fiber flax spread to Europe. However, the intermingling between fiber and oil flax in subclade C2 (Fig. 2) is consistent with the early notion that the flax spread over Europe involved both oil and fiber flax^1,3,16,60^.

Dating divergences among the five *Linum* groups were made with two approaches. BEAST-based analysis provided node age estimations of the maximum clade credibility tree, as illustrated in Fig. 3. When rooted to pale flax, presumably with 10,000 years of flax domestication, the divergences of indehiscent and winter flax from oil flax were 7117 and 5104 years BP, respectively. As mentioned above, no specific clade or branch dominant for fiber flax existed, but early fiber flax (after flax spread to Europe) was estimated to occur 4245 years BP. The more significant node, with an age estimate of 5779 years, was on the two subclades of oil, fiber and winter flax. This age estimate for flax spreading to Europe is compatible with the dating of the archaeological finds in southwest Germany with larger flax seeds in the early phase of the Late Neolithic (6000 years BP)^18^. It is worth noting that the root age of 10,000 years assumed here for oil flax was older than the earliest archaeological finds of cultivated flax in Tell Ramad, Syria, with a dating of roughly 9000 years BP^2^, but “Ramad certainly does not mark the beginning of flax cultivation^2^.” Also, the assumed root age is within the 10,000 to 12,000 years of the other Neolithic Southwest Asian founder crops like wheat and barley in the Near East^4^.

Dating the *Linum* group divergences through the SMCPP-based demographic inferences also provided useful estimates of splitting time between any two *Linum* groups, as illustrated in Table 3. Interestingly, the results of divergence in years for four assayed pairs of the five *Linum* groups were compatible with those obtained from the BEAST-based analysis. For example, the estimated separation of oil flax from pale flax was 7461 years ago, but with a range of 5027 to 12,092 years. Similarly, the separations of indehiscent and winter flax from oil flax were estimated to occur 7093 and 4905 years BP, while compared to the BEAST-based estimates of 7117 and 5104 years, respectively. However, the estimate of splitting time between fiber and oil flax (or 5407 years BP) seemed to match well with the separation of the two subclades for flax spreading to Europe (or 5779 years BP), as the BEAST-based MCC trees (Figs. 2 and 3) had no specific clade or branch dominantly for fiber flax to compare. It is worth noting that the divergence estimates from both approaches were not significantly different as these age estimates had large standard deviations. Also, all the dating inferences are scalable. For example, if the root age is set as 9000 or 11,000 years BP instead, the estimated node ages (Fig. 3) or splitting times (Table 3 or Fig. 5) can be adjusted by a multiplication of 0.9 (= 9000/10,000 years) or 1.1, respectively. Despite these compatible comparisons and compatible results with those reported^38^, however, it should be mentioned that SMCPP-based dating had many estimates that were not convergent for many chromosomes and yielded estimates with large standard deviations (Table 3). These issues may have well reflected the impacts of violating the gene flow assumption with detected mixture events (see Fig. 4) and revealed a part of weakness for the dating approach.

**Fig. 6:**
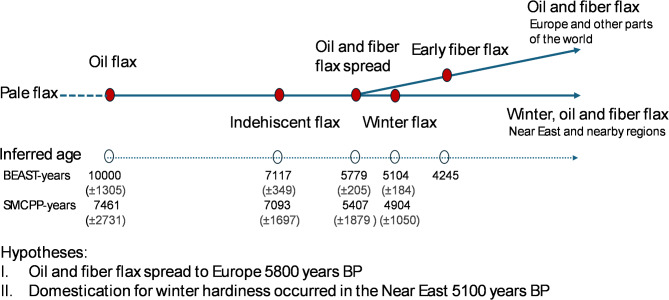
Inferred flax domestication processes with divergence dating information and new hypotheses. The standard deviations are given in parentheses for the inferred years of various domestication events by BEAST and SMCPP, assuming 10,000 years of flax domestication.

To better summarize our current understanding of flax domestication history, we illustrated the inferred flax domestication processes with divergence dating information in Fig. 6. Clearly, the picture of flax domestication processes is not complete, nor comprehensive, but it can serve as a draft genetics-inked picture to be painted and refined with further research. To facilitate future research, we also formulated and presented two new hypotheses (Fig. 6). The first hypothesis is that oil and fiber flax spread to Europe 5800 years BP. This hypothesis reflected the new genetic signals acquired from this study, but as pointed out above, the time of flax spreading is compatible with the archaeological finds^18^. However, some questions remain: did oil flax spread first alone or together with fiber flax? and if it is the former, when did fiber flax spread to Europe? One extra genetic signal acquired here was the finding of the earliest fiber flax from Czech Republic inferred with the age of 4245 years (Fig. 3). The second hypothesis is that domestication for winter hardiness occurred in the Near East 5100 years BP. This hypothesis was derived based on three aspects of the genetic signal: (1) the countries of origin for the assayed winter flax samples, in which 7 (out of 14) samples originated from the Near East (Fig. 3); (2) the compatible genetic signals of splitting times for winter flax inferred by two different approaches (Fig. 3; Table 3); and (3) the close relations of winter flax with oil flax in subclade D (Fig. 3). This hypothesis also had the support from Heer’s conclusion that flax was a winter crop in Egypt^6,21^. However, the reasoning cannot explain the genetic transitions of perennial and/or winter type of pale flax into winter annual and further into summer annual cultivated flax, which deserves further research. It is possible that the assayed winter flax samples carried the genetic signatures largely of frost hardiness and not much of ability to vernalize. Also, it does not provide extra insight into flax spread north to Europe^20^. An improved frost hardiness was likely needed for cultivated flax spreading to Europe 6000 years BP^18^. Surprisingly, little genetic signal existed for frost hardiness improvement in subclade C, which was predominantly composed of oil and fiber flax (Fig. 3), except the winter flax sample w5 from Argentina, a descendent of cultivated flax in Italy (Table 1). In spite of these inferences, these two hypotheses still need to be tested with more informative genetic data and archaeological finds. More importantly, further genetic inferences should be made by incorporating early agro-ecosystems^61^, agricultural use of domestication traits^62^, and/or cultural context^17^.

As discussed above, our genetic inferences did not provide clear genetic signals for the initial domestication of fiber flax. However, some relevant information is worth mentioning. First, the finding of fiber flax intermingled genetically with oil and winter flax implied a possibility that the initial domestication for fiber, like winter hardiness, occurred in the Near East. Second, the previous inferences based on the *sad*2 locus suggested that flax was domesticated first for oil, not fiber, use^11,12^, although the debate for the first domestication use remains open, as twined fabrics dated to 9000 years BP were also found in the southern Levant^14,62^. Third, some genetic signals existed that the earliest fiber flax after flax spread to Europe was represented by the fiber cultivar “Zakar” (or sample f12) that were collected on March 24, 1977, from Czech Republic and it was dated 4245 years BP (Fig. 3), although this dating was 1300 years later than those suggested by archaeological records of fiber flax in Europe (or 5500 BP)^16^.

This study also displayed some weaknesses worth mentioning. First, the assayed samples of indehiscent flax did not reveal clear genetic signals for the initial domestication region for capsular dehiscence, which largely reflected the weakness in the geographic sampling of indehiscent flax. However, considering the finding that domestication for capsular dehiscence was much earlier than that for winter hardiness (Fig. 3), one could also speculate that the originating region for indehiscent flax, like winter flax, was in the Near East. Second, our sampling of cultivated flax had inadequate regional representation, particularly from the region of Indian subcontinent in which cultivated flax is genetically distinct^63^. Also, our grouping of cultivated flax was not fully exclusive, as some samples could represent different groups. For example, the indehiscent flax sample CN101424 could also be considered as fiber flax. Some winter flax samples such as CN96960 and CN98509 could be deemed as oil flax, as their oil contents were also high. Non-exclusive *Linum* grouping could affect the reported genetic inferences such as those of mixture events (Fig. 4). Third, our sample size for various groups of cultivated flax was relatively small, so our efforts using advanced inference tools such as dadi^64^ and moments^65^ to infer flax spreading from the Near East to Europe were not fruitful. With the technical advances in genetic inferences of population demographic history^66,67^, it is feasible to infer flax spreading to Europe and other parts of the world, if sufficiently large samples of various domestication groups with better geographic coverage are genotyped^35^. Such inferences will also allow for better resolutions in the estimations of *Linum* group divergences. Fourth, our study also suffered from the lack of sufficient geographic coverage of pale flax samples, particularly in many countries in the Near East and North Africa. Currently, there are no pale flax germplasm accessions collected from the regions of Jordan, Syria, Iraq, Iran, and northern Africa, and large geographic gaps exist in the species distribution^22^. Without pale flax collections from those regions, it is impossible for genetic inferences to generate a complete picture of flax domestication processes.

## Conclusions

This study revealed that pale flax had the largest nucleotide diversity, followed by indehiscent, winter, oil and fiber cultivated flax. Pale flax seemed to be under population contraction, while the other four domestication groups were under population expansion after bottleneck. Multiple mixture events existed among the five *Linum* groups. There were two separate phylogenetic clades for oil, fiber and winter flax. One clade consisted of all three domestication groups, but predominantly winter flax, and these samples mainly originated in the Near East and nearby regions. The other clade mainly had oil and fiber flax originating from Europe and other parts of the world. Dating genetic divergences revealed that oil and fiber flax spread to Europe 5800 years BP and domestication for winter hardiness occurred in the Near East 5100 years BP.

## Electronic supplementary material

Below is the link to the electronic supplementary material.

Supplementary Material 1

## Data Availability

The raw sequences were deposited into NCBI’s SRA database under BioProject ID of PRJNA1106517 in April 2024.
