# Supplementary material for: Flax domestication processes as inferred from genome-wide SNP data
Source: Sci Rep. 2025 Mar 13;15:8731. doi: 10.1038/s41598-025-89498-9 (PMC11906640; doi:10.1038/s41598-025-89498-9)

Yong-Bi Fu (2025) Flax domestication processes as inferred from genome-wide SNP data. Scientific Reports (10.1038/s41598-025-89498-9)

Supplemental materials:

Supplemental Tables

Table S1. Summary of MiSeq sequence reads before trimming, after trimming, and after alignment for 93 *Linum* samples.

Table S2. SNP counts across 15 flax chromosomes for all 93 samples and five *Linum* groups (pale flax, oil flax, fiber flax, winter flax and indehiscent flax).

Supplemental Figures

Figure S1. Patterns of allelic frequency distributions for all 93 samples (All) and five *Linum* groups (pale flax, oil flax, fiber flax, winter flax and indehiscent flax).

Figure S2. The phylogenetic tree of 93 *Linum* samples representing pale flax and four domestication groups of cultivated flax obtained by the RAxML program. Sample label is shown (see Table 1). This tree has the same topology as the maximum clade credibility trees from the BEAST program (Figure 2).

Figure S3. The NeighborNets of 93 *Linum* samples representing pale flax and four domestication groups of cultivated flax obtained by the SplitsTree4 program. Two samples of indehiscent flax (d9 and d10) are highlighted in red, as they are close to three other groups of cultivated flax. The NeighborNets show the same patterns of phylogenetic inferences as the maximum clade credibility trees from the BEAST program (Figure 2).

Figure S4. The maximum clade credibility (MCC) trees of 54 selected *Linum* samples representing pale flax and four domestication groups of cultivated flax obtained by the BEAST program. These two MCC trees are the same as Figure 3, but are presented here to illustrate their node height estimates (**A**) and node height_95%_HPD estimates (**B**) to support the dating inferences.

Figure S5. The maximum likelihood trees of five *Linum* groups (b=pale flax, d=indehiscent flax, w=winter flax, f=fiber flax, and o=oil flax) with the mixture events, inferred from TreeMix with migration edges from m=1 to 10.

Table S1. Summary of MiSeq sequence reads before trimming, after trimming, and after alignment for 93 *Linum* samples.

Sample sequence label	MiSeq run	fastq paired reads	Trimmed fastq paired reads	Percent trimmed paired reads	Total reads in bam file	Total mapped reads in bam file	Percent mapped reads in bam file
CN107257_S1	1	1,105,377	750,501	67.90	1,518,098	1,497,040	98.61
CN19021_S2	1	1,196,964	893,254	74.63	1,824,833	1,783,942	97.76
CN97606_S3	1	1,969,447	1,381,736	70.16	2,842,570	2,759,675	97.08
CN100852_S4	1	1,631,368	1,142,330	70.02	2,357,928	2,280,558	96.72
CN98833_S5	1	1,348,016	970,743	72.01	2,001,641	1,939,247	96.88
CN97605_S6	1	1,326,466	929,059	70.04	1,886,887	1,855,385	98.33
CN100837_S7	1	1,195,000	866,515	72.51	1,753,140	1,726,770	98.50
CN18974_S8	1	1,094,525	785,091	71.73	1,589,211	1,569,042	98.73
CN100917_S9	1	1,075,470	731,397	68.01	1,500,594	1,460,108	97.30
CN98178_S10	1	1,623,710	1,168,435	71.96	2,359,315	2,333,903	98.92
CN97004_S11	1	1,649,849	1,183,269	71.72	2,386,145	2,363,474	99.05
CN96960_S12	1	1,683,349	1,179,828	70.09	2,382,212	2,345,542	98.46
CN113643_S13	1	1,407,745	978,163	69.48	1,976,042	1,954,857	98.93
CN52732_S14	1	1,470,047	1,017,103	69.19	2,045,903	2,032,156	99.33
CN107258_S15	1	609,972	436,914	71.63	888,964	872,696	98.17
CN19022_S16	1	1,177,049	829,884	70.51	1,679,809	1,657,384	98.67
CN97205_S1	2	1,831,532	1,308,890	71.46	2,658,087	2,607,700	98.10
CN97756_S2	2	1,915,820	1,440,727	75.20	2,916,228	2,870,824	98.44
CN96846_S3	2	1,898,858	1,435,546	75.60	2,897,852	2,859,869	98.69
CN98283_S4	2	1,969,443	1,459,498	74.11	2,951,613	2,891,787	97.97
CN98509_S5	2	1,529,374	1,173,128	76.71	2,365,642	2,337,580	98.81
CN97009_S6	2	1,175,221	886,644	75.44	1,793,192	1,766,611	98.52
CN97102_S7	2	1,268,873	970,644	76.50	1,979,993	1,910,931	96.51
CN100910_S11	2	1,847,132	1,421,743	76.97	2,962,050	2,839,986	95.88
CN101086_S12	2	144,600	101,087	69.91	203,967	201,661	98.87
CN33399_S13	2	1,635,844	1,239,165	75.75	2,505,452	2,467,179	98.47
CN97769_S14	2	2,011,566	1,516,807	75.40	3,075,997	3,029,355	98.48
CN113606_S15	2	543,837	425,541	78.25	872,122	845,888	96.99
CN19003_S16	2	1,483,892	1,158,989	78.10	2,356,311	2,314,237	98.21
CN18989_S1	3	1,762,341	1,226,030	69.57	2,508,012	2,430,854	96.92
CN98935_S2	3	2,506,286	1,923,153	76.73	3,953,485	3,840,129	97.13
CN96915_S3	3	1,454,211	1,069,153	73.52	2,156,011	2,126,202	98.62
CN100829_S4	3	1,506,017	1,102,014	73.17	2,281,678	2,187,535	95.87
CN113632_S5	3	1,479,414	1,058,195	71.53	2,164,377	2,105,881	97.30
CN107293_S6	3	1,630,364	1,168,126	71.65	2,367,628	2,333,693	98.57
CN101265_S7	3	1,341,658	985,189	73.43	2,045,474	1,966,664	96.15
CN101174_S9	3	2,311,354	1,734,132	75.03	3,659,791	3,436,653	93.90
CN101292_S10	3	463,939	315,971	68.11	667,220	630,346	94.47
CN101405_S11	3	1,940,939	1,358,221	69.98	2,771,587	2,697,713	97.33
CN113622_S12	3	1,658,402	1,213,021	73.14	2,500,461	2,411,854	96.46
CN19023_S13	3	1,605,163	1,151,067	71.71	2,355,204	2,299,901	97.65
CN101160_S14	3	396,436	266,654	67.26	546,703	532,966	97.49
CN97473_S15	3	1,251,437	1,064,043	85.03	2,141,878	2,103,018	98.19
CN101388_S15	3	1,257,078	889,764	70.78	1,838,039	1,765,513	96.05
CN97768_S16	3	1,552,348	1,103,090	71.06	2,264,745	2,196,196	96.97
CN101171_S1	4	1,306,688	974,830	74.60	1,987,278	1,930,155	97.13
CN113618_S2	4	1,962,585	1,511,931	77.04	3,083,558	3,013,772	97.74
CN113608_S3	4	1,710,623	1,334,163	77.99	2,729,061	2,660,658	97.49
CN100828_S4	4	1,600,570	1,232,106	76.98	2,483,561	2,437,125	98.13
CN18991_S5	4	2,509,651	1,999,774	79.68	4,025,978	3,988,316	99.06
CN100832_S6	4	1,280,106	986,658	77.08	2,007,817	1,968,242	98.03
CN113640_S7	4	1,098,679	853,048	77.64	1,737,377	1,700,791	97.89
CN113634_S8	4	1,431,239	1,128,176	78.83	2,298,507	2,245,066	97.67
CN101111_S9	4	288,456	179,273	62.15	365,732	357,278	97.69
CN101392_S10	4	1,700,244	1,334,775	78.50	2,727,427	2,660,184	97.53
CN96848_S11	4	890,728	652,122	73.21	1,318,022	1,297,016	98.41
CN101397_S12	4	1,978,314	1,542,942	77.99	3,125,674	3,074,633	98.37
CN113633_S13	4	1,470,104	1,151,425	78.32	2,314,031	2,291,296	99.02
CN113623_S14	4	1,206,514	937,951	77.74	1,898,937	1,868,890	98.42
CN98946_S15	4	536,115	388,592	72.48	790,490	774,298	97.95
CN96902_S16	4	1,730,907	1,391,443	80.39	2,806,561	2,708,647	96.51
CN101268_S1	5	1,494,313	1,145,481	76.66	2,308,467	2,285,447	99.00
CN113610_S2	5	1,441,048	1,189,376	82.54	2,421,510	2,371,086	97.92
CN97325_S3	5	1,379,071	1,135,600	82.35	2,318,521	2,189,305	94.43
CN113638_S4	5	1,630,350	1,346,822	82.61	2,738,149	2,685,521	98.08
T19717_S5	5	1,379,018	1,134,797	82.29	2,302,070	2,262,009	98.26
CN97888_S6	5	1,168,649	988,690	84.60	2,019,423	1,967,405	97.42
CN113637_S7	5	1,207,693	969,813	80.30	1,997,906	1,931,613	96.68
CN113635_S8	5	1,251,679	1,052,856	84.12	2,142,514	2,089,932	97.55
CN113641_S9	5	2,263,934	1,931,877	85.33	3,944,809	3,824,110	96.94
CN113636_S10	5	1,438,280	1,245,844	86.62	2,523,080	2,478,929	98.25
CN113603_S11	5	1,292,966	1,119,803	86.61	2,302,357	2,223,225	96.56
CN113630_S12	5	1,610,119	1,352,709	84.01	2,749,045	2,687,273	97.75
CN113627_S13	5	1,118,339	954,738	85.37	1,934,347	1,892,129	97.82
CN101614_S14	5	1,209,133	996,026	82.38	2,033,856	1,973,126	97.01
CN101233_S15	5	1,131,702	940,014	83.06	1,913,125	1,871,210	97.81
CN98479_S16	5	330,004	272,555	82.59	556,036	541,854	97.45
CN113626_S1	6	1,447,993	1,179,368	81.45	2,398,025	2,346,931	97.87
CN113617_S2	6	1,787,161	1,529,901	85.61	3,098,392	3,043,455	98.23
CN113628_S3	6	1,649,332	1,383,976	83.91	2,816,193	2,756,430	97.88
CN98256_S4	6	2,018,169	1,638,727	81.20	3,290,011	3,259,043	99.06
CN101424_S5	6	1,263,908	1,063,874	84.17	2,150,349	2,114,338	98.33
CN98475_S6	6	1,177,363	983,473	83.53	1,979,851	1,930,026	97.48
CN97436_S7	6	1,345,566	1,143,011	84.95	2,316,739	2,277,221	98.29
CN97473_S8	6	1,689,657	1,223,197	72.39	2,538,695	2,439,272	96.08
CN97871_S8	6	1,374,296	1,133,752	82.50	2,289,489	2,244,538	98.04
CN98986_S9	6	1,281,117	1,046,306	81.67	2,111,097	2,064,039	97.77
CN101017_S10	6	109,180	80,290	73.54	162,033	158,770	97.99
CN101245_S11	6	1,555,767	1,302,629	83.73	2,633,668	2,589,881	98.34
CN113620_S12	6	1,575,790	1,312,134	83.27	2,649,830	2,598,149	98.05
T19718_S13	6	2,073,409	1,779,884	85.84	3,601,427	3,530,695	98.04
CN101237_S14	6	1,151,084	971,417	84.39	1,958,191	1,927,578	98.44
CN113629_S16	6	1,300,119	1,130,783	86.98	2,280,401	2,231,600	97.86
Average		1,417,048	1,093,434	76.82	2,225,094	2,174,226	97.72

Table S2. SNP counts across 15 flax chromosomes for all 93 samples and five *Linum* groups (pale flax, oil flax, fiber flax, winter flax and indehiscent flax).

			Cultivated flax			
	All	Pale flax	Oil	Fiber	Winter	Indehiscent
Sample size	93	31	20	16	15	11
Chromosome						
1	1295	1075	719	547	656	802
2	872	674	303	260	296	558
3	1336	1088	576	510	590	913
4	1438	1249	839	760	842	852
5	1038	933	547	482	556	601
6	1147	983	615	509	607	758
7	961	820	525	440	519	628
8	1168	987	609	458	626	718
9	1192	970	525	423	516	777
10	1198	931	526	450	523	894
11	850	746	471	405	482	549
12	1052	850	449	435	483	692
13	888	799	477	444	486	567
14	1163	941	328	302	340	800
15	1400	1202	851	696	831	946
Total	16998	14248	8360	7121	8353	11055

Figure S1. Patterns of allelic frequency distributions for all 93 samples (All) and five *Linum* groups (pale flax, oil flax, fiber flax, winter flax and indehiscent flax).

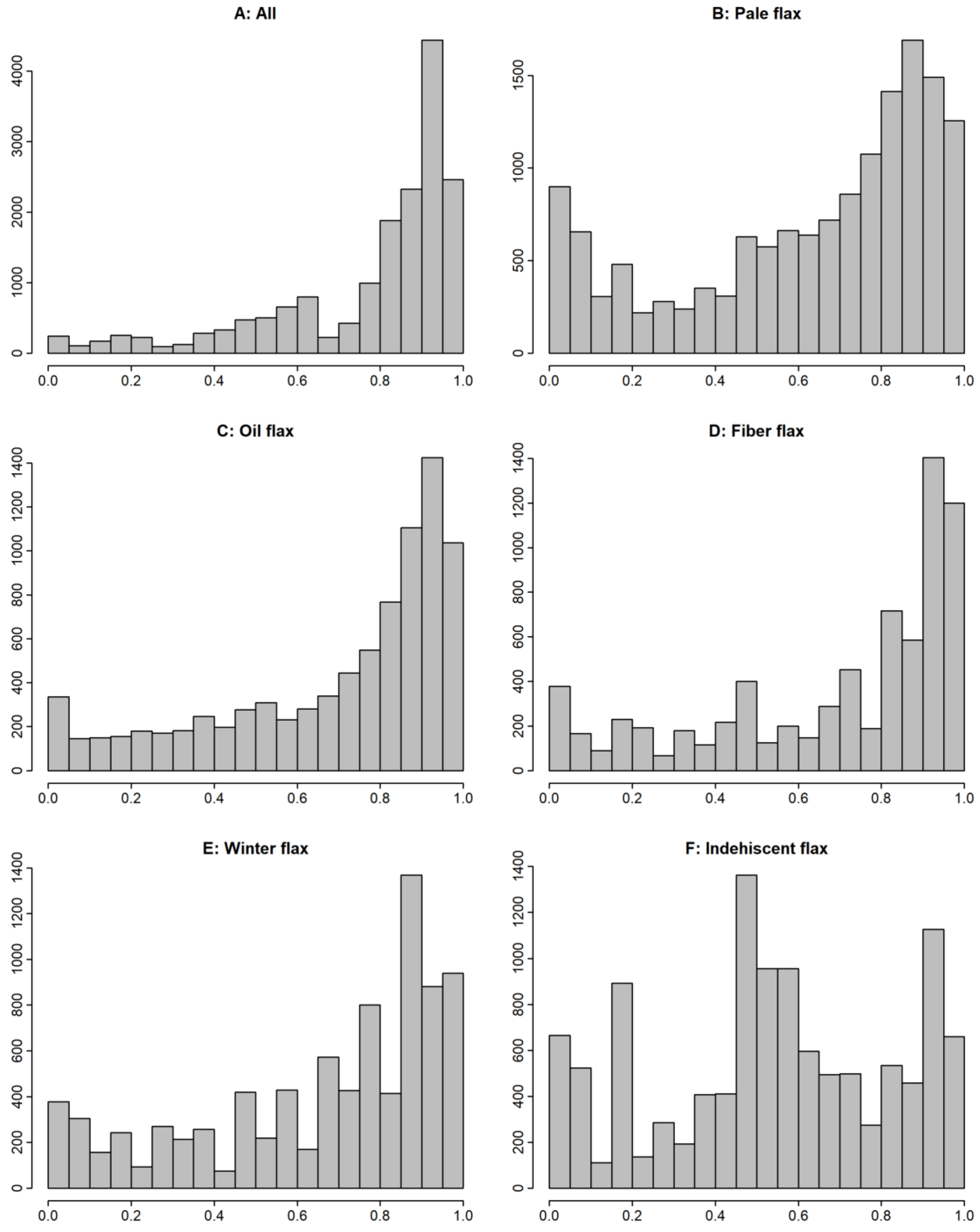

Figure S2. The phylogenetic tree of 93 *Linum* samples representing pale flax and four domestication groups of cultivated flax obtained by the RAxML program. Sample label is shown (see Table 1). This tree has the same topology as the maximum clade credibility trees from the BEAST program (Figure 2).

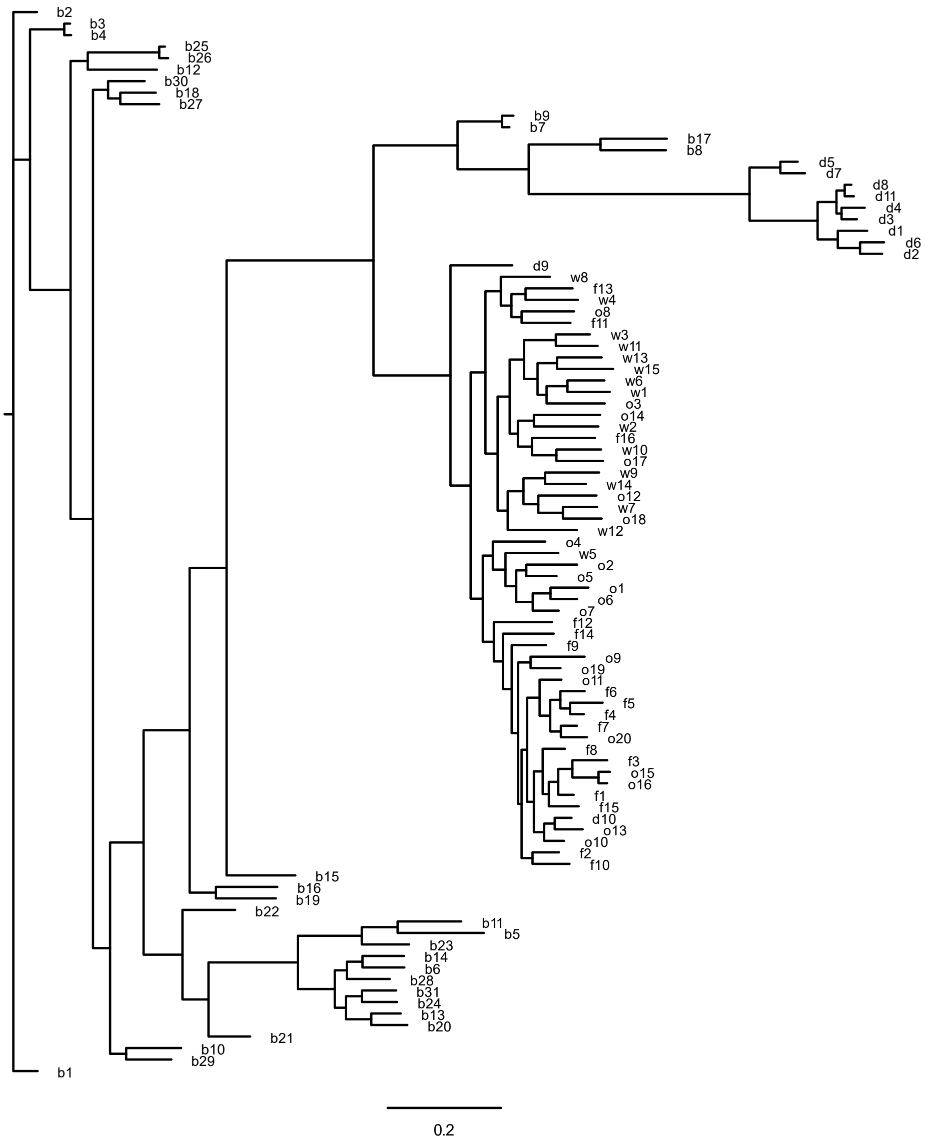

Figure S3. The NeighborNets of 93 *Linum* samples representing pale flax and four domestication groups of cultivated flax obtained by the SplitsTree4 program. Two samples of indehiscent flax (d9 and d10) are highlighted in red, as they are close to three other groups of cultivated flax. The NeighborNets show the same patterns of phylogenetic inferences as the maximum clade credibility trees from the BEAST program (Figure 2).

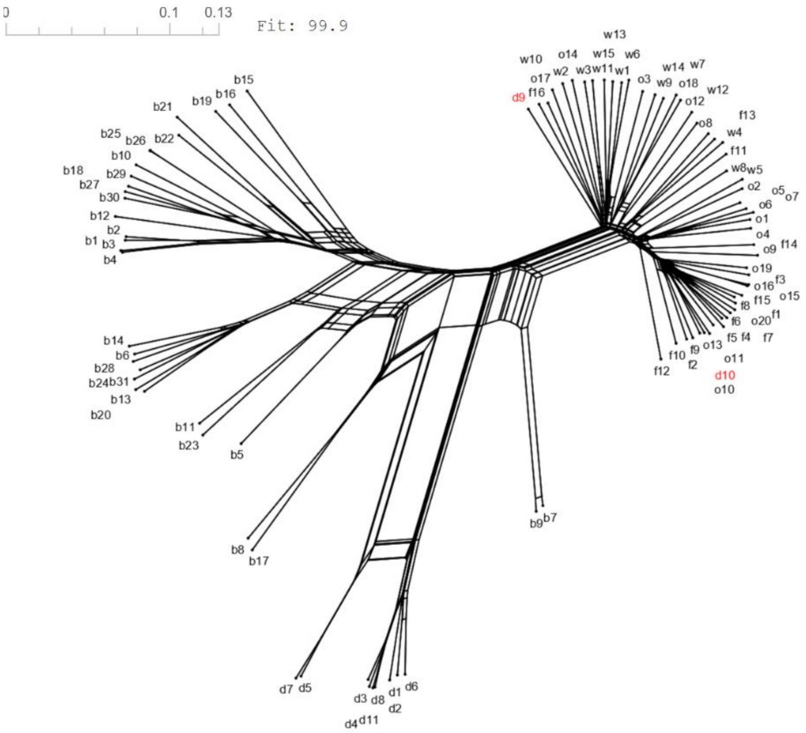

Figure S4. The maximum clade credibility (MCC) trees of 54 selected *Linum* samples representing pale flax and four domestication groups of cultivated flax obtained by the BEAST program. These two MCC trees are the same as Figure 3, but are presented here to illustrate their node height estimates (**A**) and node height_95%_HPD estimates (**B**) to support the dating inferences.

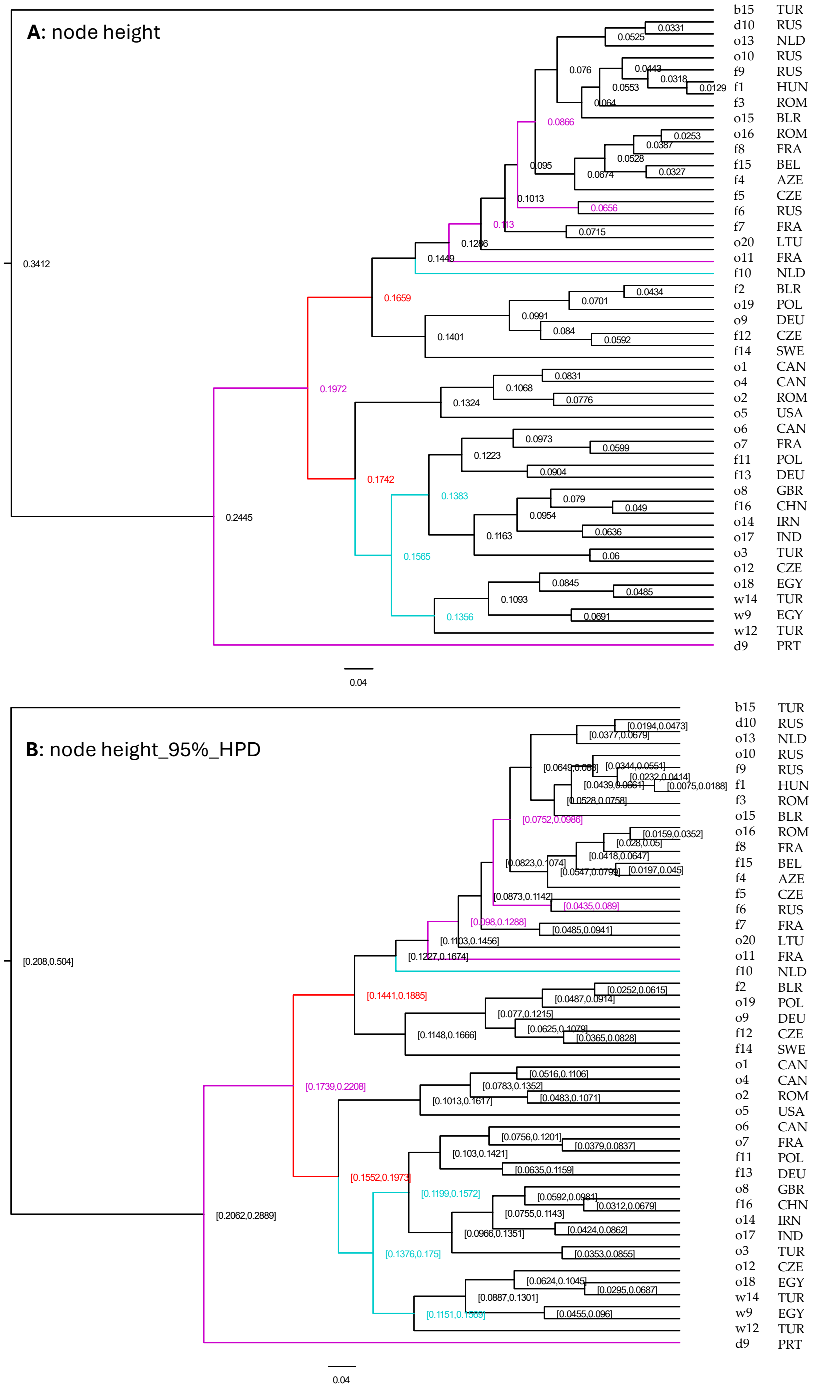

Figure S5. The maximum likelihood trees of five *Linum* groups (b=pale flax, d=indehiscent flax, w=winter flax, f=fiber flax, and o=oil flax) with the mixture events, inferred from TreeMix with migration edges from m=1 to 10.

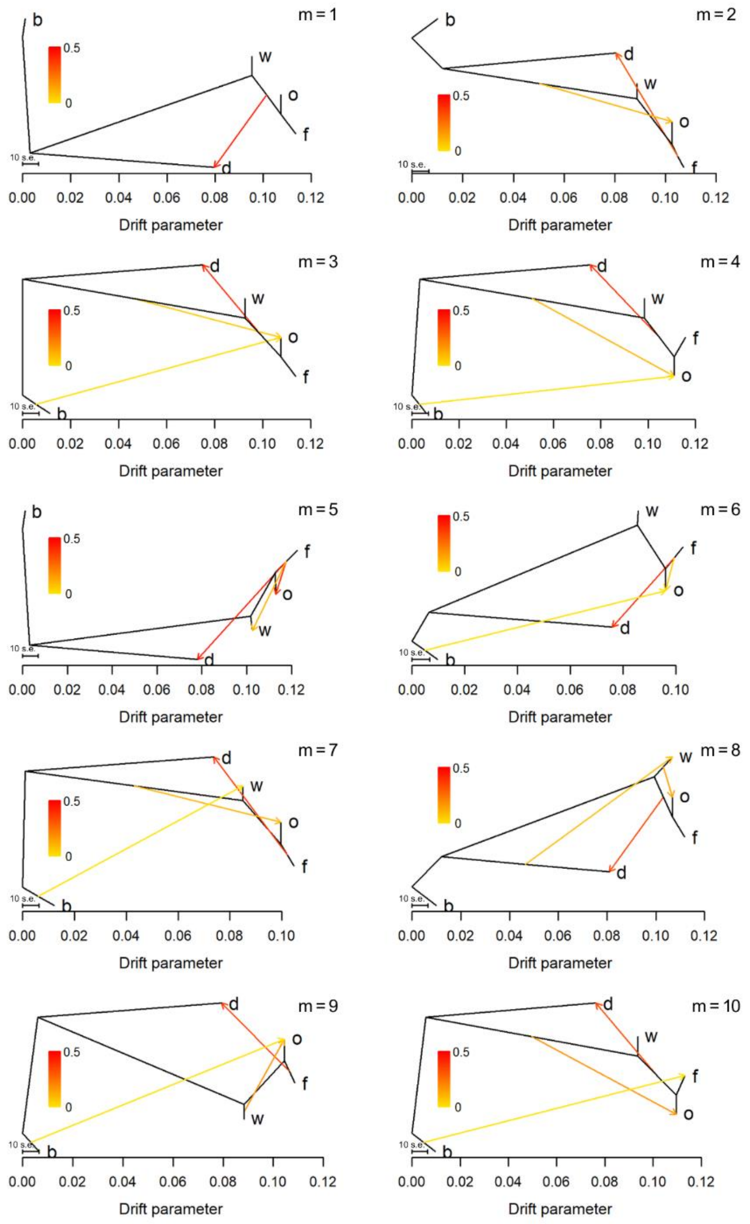

Supplement: Supplementary file 1 — Supplementary Material 1 [file 41598_2025_89498_MOESM1_ESM.pdf]
